# Diphtheria — Thermic Fever—Œsophageal Stricture

**Published:** 1879-02

**Authors:** C. M. Easton

**Affiliations:** Gardner, Ill.


### NOTES FROM PRIVATE PRACTICE.

### ARTICLE VII.

*Diphtheria.—Thermic Fever.—Œsophageal Stricture.*

1. During the late autumn, fourteen children have died of diphtheria in a locality about fifteen miles southeast of Gardner, Ill. In one family, three of four children died. In another of six, three died. In another of seven, six died.

2. One evening during the heated term of last summer, I was called to attend a lady 80 years old. The son reported that after visiting the orchard, she was seen to stagger, and was helped into the house. She joined the rest of the family at supper, and was seen to eat a little, but seemed unconscious of what she was doing. After supper, she started to reach a door, fell to the ground, and was picked up completely unconscious. I found her in a comatose condition, with stertorus breathing, pulse 140, temperature 108°. Dr. Taxis being summond to my aid, we applied ice to the head and administered Norwood's tincture of veratrum virida. She remained totally unconscious for thirty-six hours; then rallied, and was soon as well as before.

3. Mr. Thompson, a native of Ireland, came to this country when 25 years of age. At this time he swallowed, by mistake, a corrosive acid, which produced great soreness of the mouth and throat, and resulted in œsophageal stricture. Being unable to swallow solid food, and being also fond of milk and cake, he made that his exclusive diet.

When I first saw him, he told me he had lived on this cake and milk for about 55 years. His story was corroborated by his wife and daughter, and I have no doubt of its truthfulness. I was their family physician for a number of years. His wife told me she made his cake very sweet and short; he dissolved it in his milk, and had eaten this only for more than half a century. He could not and did not swallow anything as large as an apple-seed after his injury. He was a farmer, and up to within a few years of his death was able to perform laborious farm work.

This man lived to a ripe old age—something over four score years. The extreme contraction of the œsophagus, the exclusiveness of the diet, his ability to perform hard labor, with his long lease of life, are noteworthy facts.

C. M. EASTON.

GARDNER, ILL.

